# Awareness level of cancer risk factors and warning signs among adults in Mansoura District, Egypt

**DOI:** 10.1186/s12889-026-29125-y

**Published:** 2026-09-03

**Authors:** Ahmed M. Eid, Abeer M. Kamal, Asmaa Elsayed, Rahma M. Almetwaly, Basma Kamel, Naira A. Shoman, Aya M. Abdelmohsin, Sara H. Elhendawy, Yasmeen A. State, C. A. collaboration team, Abdallah Gamal, Menna S. Sellow, Nourhan R. Elbatal, Mohamed A. Abdelaziz, Ahmad M. Gewili, Mariam B. Elbialy, Nouran K. Mashaly, Rania S. Omar, Abdelrahman M. Darweesh, Abdelsmad Elhalwaty, Hager S. Elhebeshy, Moustafa Y. Nassar, Hanin Fadl, Ahmed H. Maarouf, Omar M. Eid, Sama Mahmoud Abo Younis, Ashraf Alaziz, Mokhtar M. Elkerdawey, Ahmed Mohamed Zayed, Abdel-Hady El-Gilany

**Affiliations:** 1 Faculty of Medicine, Mansoura University, Mansoura, 35516 Egypt; 2 Faculty of Medicine, Horus University - (HUE), New Damietta City, Egypt; 3 ORL Department, Faculty of Medicine, Mansoura University, Mansoura, Egypt; 4 Public Health and Preventive Medicine, Mansoura University, Al Mansurah, Egypt

**Keywords:** Knowledge, Awareness, Cancer, Risk Factors, Warning Signs, Mansoura, Egypt

## Abstract

**Background:**

Cancer remains a leading cause of mortality worldwide, with prevention and early detection dependent on the public awareness of its risk factors and warning signs. However, knowledge of cancer-related symptoms and modifiable risk factors remains limited in many populations, particularly in low- and middle-income countries. Limited research exists examining general cancer awareness in Egypt's general population.

**Objective:**

To assess public knowledge of cancer's early warning signs, risk factors, and prevention strategies in Mansoura, Egypt.

**Methods:**

A descriptive cross-sectional study was conducted at five primary healthcare facilities in Mansoura from June to August 2025. We used a validated Arabic questionnaire to assess general cancer knowledge, awareness of cancer warning signs, and knowledge of its risk factors. Descriptive statistics and non-parametric tests (Mann–Whitney U and Kruskal–Wallis) were used to analyze the data. Statistical significance was set at *p* < 0.05.

**Results:**

Three hundred ninety nine adult participants completed the questionnaire (60.7% female); most were aged 18–35 and demonstrated high general cancer knowledge, with 90.2% aware that regular check-ups aid early detection. However, awareness of specific warning signs was limited—only 33.3% recognized non-healing sores and 35.1% identified persistent cough as a warning sign. Knowledge of cancer risk factors was also inconsistent; while 89.5% identified smoking as a risk factor, only 13.5% recognized genetically modified foods as a risk factor. The median overall knowledge score was 28 out of 52. Educational attainment and association with the medical field were significant predictors of cancer knowledge across all domains (*p* < 0.001), while sex and residency showed no significant associations. The internet and social media were the primary sources of information (53.9%).

**Conclusion:**

While general cancer awareness was moderately high, knowledge of specific warning signs and modifiable risk factors remains limited. Targeted educational interventions, particularly among non-medical populations and those with lower educational attainment, are essential to improving cancer prevention and early detection.

## Introduction

Cancer is a serious disease characterized by uncontrolled cell growth that may arise in any part of the body and present either localized or metastatic disease. According to the National Cancer Institute (NCI), the most common cancers are lung, breast, colorectal, and prostate [1]. Cancer remains one of the leading causes of mortality worldwide, with nearly 10 million deaths reported in 2020 [2]. Moreover, the global cancer burden continues to rise, with the World Health Organization (WHO) projecting a 70–77% increase in new cases over the next two decades [3, 4]. Collectively, these figures underscore the substantial burden cancer imposes on healthcare systems, patients, and families.

Most cancers arise from potentially modifiable risk factors, including tobacco use, alcohol consumption, unhealthy diet, obesity, physical inactivity, infections, pollution, iodine intake, vitamin D deficiency, sunlight exposure, radiation, and hormone replacement therapy [5, 6]. Awareness of these factors may improve prognosis [7] and support prevention efforts. The WHO estimates that 30–50% of cancers could be prevented through risk-factor avoidance and increased awareness. For example, a study reported that modifying smoking behavior reduces the risk of lung cancer [8]. Despite advances in cancer diagnosis and treatment [9], early detection remains challenging because patients often delay seeking medical care. This delay adversely affects prognosis and treatment outcomes [10], particularly in low- and middle-income countries, including those in the Arab region [11].

Awareness of cancer risk factors and warning signs remains limited in the general population. A 2011 study in Tehran found that only 18.8% of participants had high awareness [12]. Recognition of warning signs is similarly inconsistent; a 2020 study reported that signs such as a "lump" were moderately recognized, whereas changes in bowel habits were poorly recognized [13]. Moreover, perceptions of specific risk factors vary substantially. For instance, although 88.5% of participants in one study identified smoking as a risk factor, only 11.9% recognized poor nutritional habits, such as inadequate intake of fruits and vegetables, as a cancer risk [14]. Likewise, a study in Jordan found that more than half of the population had only fair knowledge of colorectal cancer, while fewer than 20% demonstrated poor knowledge [15].

In the Egyptian context, several studies have assessed awareness of specific cancers, primarily breast, colorectal, and cervical cancer [16–18]. However, these investigations have been largely disease-specific, resulting in a fragmented understanding of cancer within the broader population. Although awareness of highly publicized risks, such as smoking, and of certain localized cancers is relatively well established, knowledge of general warning signs and modifiable lifestyle-related risk factors remains insufficient. This underscores the need to move beyond disease-specific education and toward comprehensive, population-level health literacy initiatives that strengthen cancer prevention and early detection. Limited research exists regarding general awareness of cancer’s early warning signs, symptoms, risk factors, and prevention. Addressing this gap is essential for understanding public perceptions of cancer, which constitutes the primary aim of this study.

## Materials and methods

### Study design and settings

A descriptive cross-sectional study with an analytical component was conducted at five primary healthcare facilities in Mansoura. Adult participants were included, and data were collected from June to August 2025.

### Study sampling and sample size

The sample size was determined using the formula for estimating a single population mean with an expected standard deviation of 10.15 for overall cancer knowledge, as reported in a previous study [19]. Based on the t-distribution, 331participants were required to achieve a 95% confidence level and a margin of error of ± 1.1. This calculation was performed using the Statulator online calculator (https://statulator.com/SampleSize/ss1M.html). Participants were recruited from five of the 51 primary healthcare facilities in Mansoura based on high outpatient volume to reach the calculated sample size within the available data-collection period, using a volunteer student-led collection team. Facilities were chosen to represent both urban (two facilities) and rural (three facilities) areas within the Mansoura district, using convenience sampling within each center via paper-based questionnaires. The sample was allocated proportionally according to the number of patients in each center. Adults aged 18 years or older who provided informed consent were included, while those who declined participation were excluded.

### Data collection instrument

Data were collected using a validated Arabic questionnaire adapted from previous studies, which demonstrated good validity and reliability [19, 20]. The questionnaire was reviewed to ensure the clarity and appropriateness of the items for the Egyptian context. A pilot study involving 30 adults was conducted to assess reliability, and participants were excluded from the final analysis. The instrument comprised four sections: (1) sociodemographic characteristics, including age, sex, educational level, employment status, area of residence, association with the medical field, and previous attendance at cancer awareness campaigns; (2) general cancer knowledge (five items); (3) knowledge of cancer warning signs (nine items); and (4) knowledge of cancer risk factors. Each item had predefined response options and was scored as correct or incorrect according to an established answer key. Correct responses were assigned two points, whereas incorrect and “do not know" responses received zero points. The total knowledge score ranged from 0 to 52, with higher scores indicating greater cancer-related knowledge. Reliability testing in this study showed excellent internal consistency, with a Cronbach's alpha of 0.92 for the overall scale. Subscale reliability was also high, with Cronbach's alpha values of 0.96 for general cancer knowledge and 0.92 for cancer risk factors.

### Ethical consideration

Ethical approval was obtained from the Institutional Review Board (IRB) Committee of the Faculty of Medicine at Mansoura University (R.25.05.3204). The study complied with the ethical principles of the 1964 Declaration of Helsinki and its subsequent amendments. All participants provided voluntary informed consent before participating. The survey included an "agree" option with full informed consent details, which eligible participants were required to select before beginning the questionnaire. Anonymity and confidentiality were maintained throughout the study.

### Statistical analysis

Data were entered, checked for inconsistencies, and analyzed using the Jamovi program (version 2.3.28 for Windows). Descriptive statistics were reported as frequencies and percentages. The Kolmogorov–Smirnov test was used to assess normality, with all categories yielding *p*-values below 0.01, indicating a non-normal distribution. Quantitative data were summarized as medians and interquartile ranges (Q1-Q3). The Mann–Whitney U test and Kruskal–Wallis test were used to compare attitude scores across variables. Statistical significance was set at *p* < 0.05.

## Results

A total of 399 participants completed the questionnaire. Of these, 60.7% were female, and 58.1% were aged between 18 and 35 years. The majority resided in rural areas (67.9%) and did not report chronic diseases (70%). More than half (52.6%) had attained secondary or diploma-level education, and fewer than one-third were associated with the medical field (24.5%). Slightly more than half (55.6%) reported that their family income was barely sufficient. The primary sources of cancer information were the internet and social media (53.9%), followed by awareness programs (28.3%) (Table 1).

**Table 1:** Sociodemographic characteristics of the participants and their cancer knowledge (*N* = 399)

Variable	Category	N(%)
Sex	Female	242 (60.7%)
	Male	157 (39.3%)
Age Category (Min–Max: 18–63 years)	Young Adult (18–35)	232 (58.1%)
	Middle-Aged (36–55)	145 (36.3%)
	Senior (56 +)	22 (5.5%)
Residency	Rural	271 (67.9%)
	Urban	128 (32.1%)
Having a chronic illness	No Chronic Illness	279 (70.0%)
	Multiple Chronic Conditions	64 (16.0%)
	Cardiovascular Diseases	44 (11.0%)
	Diabetes	12 (3.0%)
Educational Level	Uneducated	50 (12.5%)
	Secondary or Diploma	210 (52.6%)
	University/Post gradute	139 (34.9%)
Association with the Medical/Health Field	No Association	301(75.4%)
	Student	43 (10.7%)
	Employee	55 (13.8%)
Family Income	Not enough	137 (34.3%)
	Barely enough	222 (55.6%)
	Enough and can save	40 (10.0%)
Knowledge Scores [Median (min–max)]	General Knowledge Score	8.0 (6.0–10.0)
	Awareness of Warning Signs	8.0 (4.0–12.0)
	Knowledge of Risk Factors	12.0 (10.0–18.0)
	Total Score	28.0 (22.0–36.0)
Source of Cancer Information*	Internet/Social Media	215 (53.9%)
	Friends/Relatives	125 (31.3%)
	Awareness Programs	113 (28.3%)
	Television/Newspapers	101 (25.3%)
	Other	7 (1.8%)

^*^Participants were allowed to choose multiple sources

Participants showed high knowledge of general cancer information. About 90.2% knew that regular medical check-ups help detect cancer early, and over 75% understood the importance of breast self-examination. Knowledge levels differed significantly by education and connection to the medical field. More than 70% of participants identified a lump in the breast or other organs as a cancer warning sign, while 58.9% recognized unexplained weight loss. Only one-third were aware that a non-healing ulcer is a warning sign, and 35.1% identified persistent cough or hoarseness as a warning sign. Knowledge of cancer warning signs varied significantly by education level and by association with the medical field (Tables 2 and 3).

**Table 2:** Relationship between socio-demographic characteristics and total knowledge score and its sub-components (*N* = 399)

	General Cancer Knowledge	Knowledge of Cancer Warning Signs	Knowledge of Cancer Risk Factors	Overall Cancer Knowledge
Sex				
Male	8 (6.00—10.0)	8 (4.00—12.0)	12 (8.00—18.0)	28 (20.00—36.0)
Female	8 (6.00—10.0)	8 (4.00—12.0)	12 (10.00—16.0)	30 (22.00—36.0)
*P*-value	0.138	0.187	0.945	0.279
Residency				
Rural	8 (6.00—10.0)	8 (4.00—12.0)	12 (10.00—16.0)	28 (22.00—36.0)
Urban	8 (6.00—10.0)	8 (4.00—14.0)	12 (10.00—18.0)	29 (21.50—36.0)
*P*-value	0.913	0.726	0.708	0.824
Educational Level				
Uneducated	7 (4.00—8.00)	8 (4.00—13.50)	12 (8.50—16.00)	29 (18.00—35.50)
High school/Diploma	8 (6.00—10.00)	8 (2.50—10.00)	12 (8.00—16.00)	28 (20.00—33.50)
University	10 (8.00—10.00)	10 (6.00—16.00)	14 (10.00—18.00)	34 (26.00—40.00)
*P*-value	< 0.001*	< 0.001*	< 0.001*	< 0.001*
Association with the medical field				
No Association	8 (6.00—10.0)	8 (4.00—12.0)	12 (8.00—16.0)	28 (20.00—34.0)
Student	8 (8.00—10.0)	8 (6.00—14.0)	14 (12.00—18.5)	32 (26.00—40.0)
Employee	10 (8.00—10.0)	10 (6.00—16.0)	16 (12.00—18.0)	34 (28.00—41.0)
*P*-value	0.002*	< 0.001*	< 0.001*	< 0.001*
Family Income				
Not enough	8 (6.00—10.0)	8 (4.00—10.0)	12 (8.00—16.0)	28 (20.00—36.0)
Barely enough	8 (6.00—10.0)	8 (4.00—12.0)	12 (10.00—18.0)	30 (22.00—37.5)
More than enough	10 (8.00—10.0)	8 (6.00—12.0)	15 (12.00—18.0)	32 (27.50—38.5)
*P*-value	0.012*	0.09	0.11	0.027*

All numbers are presented in the form of Median (Q1-Q3). *P*-values were derived from the ˣMann-Whitney U test or ªKruskal-Wallis test. *Statistically Significant

**Table 3:** Number and percentage of correct answers across subcomponents of measured cancer knowledge (*N* = 399)

Statement	Correct n (%)
General Cancer Knowledge	
Following a balanced diet reduces the risk of cancer	293 (73.4%)
Regular medical check-ups help in the early detection of cancer	360 (90.2%)
Breast self-examination is one of the best methods for detecting breast cancer	309 (77.4%)
Continuous exercise reduces the risk of developing cancer	256 (64.2%)
Cancer is an infectious disease	295 (73.9%)
Knowledge of Cancer Warning Signs	
Unexplained weight loss	235 (58.9%)
Non-healing sores	133 (33.3%)
Unusual bleeding or discharge	209 (52.4%)
Thickening or a lump in the breast or other organs	284 (71.2%)
Difficulty in swallowing	151 (37.8%)
Indigestion	141 (35.3%)
A change in a wart or mole	157 (39.3%)
Persistent cough or hoarseness	140 (35.1%)
Change in bowel or bladder habits	208 (52.1%)
Knowledge of Cancer Risk Factors	
Smoking	357 (89.5%)
Air pollution	301 (75.4%)
Eating genetically modified foods	54 (13.5%)
Unbalanced diet (low intake of vegetables and fruits)	220 (55.1%)
Drinking alcohol	286 (71.7%)
Hepatitis B virus infection	188 (47.1%)
Overweight or obesity	181 (45.4%)
Lack of physical activity	219 (54.9%)
Exposure to ionizing radiation (e.g., dental or CT scans)	196 (49.1%)
Exposure to ultraviolet radiation (sunlight)	155 (38.8%)
Genetic and hereditary factors	244 (61.2%)
Advancing age	158 (39.6%)

Knowledge of cancer risk factors varied among participants. Approximately 89.5% identified smoking and 75.4% identified air pollution as risk factors. Only 13.5% recognized eating genetically modified food as a risk factor. Differences in knowledge were significant by education level and by association with the medical field (Tables 2 & 3). The median overall knowledge score was 28, indicating a relatively low level of cancer knowledge, particularly regarding warning signs and risk factors. Overall knowledge was affected by education, connection to the medical field, and family income, but not by sex or residency (Tables 1 & 2).

## Discussion

Cancer is widely recognized as a serious disease and remains one of the leading causes of mortality worldwide, continuing to pose a significant public health challenge [21]. This persistent burden is often attributed to insufficient public awareness of cancer, its symptoms, and the risk factors contributing to its development [22]. Enhancing public knowledge is therefore essential, as increased awareness has been associated with improved recognition of warning signs and, in some cases, greater participation in preventive practices such as cervical cancer screening [23]. To date, no studies have specifically examined this issue in Mansoura, a major Egyptian city, underscoring the importance of the present study. A validated Arabic questionnaire from previous research was employed to assess general cancer knowledge, risk factors, and warning signs [19, 20]. The findings indicate that participants' overall cancer knowledge was moderate, with a median score of 28 out of 52. Although general knowledge was relatively high, awareness of cancer warning signs and risk factors was limited. This suggests that while participants are generally familiar with cancer, they may lack the specific knowledge necessary for early detection and prevention.

Our study found that age had no significant effect on any cancer knowledge domain, consistent with Sabi et al. [19], who reported no significant association between age and knowledge of cancer warning signs. However, this finding contrasts with Al-Azri et al. [24], who reported greater knowledge of warning signs among older participants. Similarly, Algamdi et al. [25] identified educational level and previous family history of cancer, rather than age, as determinants of knowledge of cancer risk factors. In contrast, Ahmed et al. [26] reported slightly better knowledge among individuals older than 45 years than among those aged 18–24 years. These discrepancies may reflect the influence of accumulated life experience on knowledge of a common disease such as cancer.

Sex was not significantly associated with cancer knowledge, with females demonstrating higher knowledge. This may reflect greater exposure to cancer awareness campaigns targeting conditions that disproportionately affect women, such as breast and cervical cancer [27]. In contrast, men generally have lower cancer knowledge and lower participation in screening, which may contribute to delayed diagnosis and higher cancer-related mortality [28]. For knowledge of warning signs, females also tended to score higher; this is consistent with Batra et al. [29], although Alanazi et al. [30] reported no significant sex-related difference. By contrast, sex was not significantly associated with knowledge of cancer risk factors, in agreement with Farsi et al. [31], but in contrast to Al Bashir et al. [32], who reported better knowledge among females. This discrepancy may be related to differences in exposure to health information or interactions with healthcare providers. Overall cancer knowledge also did not differ significantly between males and females, which contrasts with another study, which found higher overall knowledge among females [33]. These findings underscore the need to target men more effectively in cancer awareness campaigns.

Residency influenced the study findings, with approximately two-thirds of participants residing in rural areas. Knowledge scores were broadly similar between rural and urban participants across all domains. Several studies have documented meaningful rural–urban disparities in cancer knowledge, awareness, and preventive behaviors. Odukoya and Fayemi [34] found that adults in urban areas had significantly higher colorectal cancer knowledge and reported more frequent preventive practices than rural residents, with educational level exerting a strong influence in both groups. Similar patterns have been reported in Egypt, where breast cancer incidence is estimated to be 3–4 times higher in urban than in rural regions, likely reflecting differences in awareness, screening uptake, and exposure to risk factors [35]. Additional evidence from Port Said indicates substantial knowledge gaps among rural populations regarding colorectal cancer risk factors and screening [36]. Research on breast cancer stigma among Egyptian medical students further shows that rural participants exhibit lower awareness and greater stigma than their urban counterparts [37]. Although based in the United States, findings from the SEER-CAHPS study also highlight broader rural–urban disparities in cancer care access and patient experience, offering relevant insight into similar challenges in low- and middle-income contexts [38]. These disparities identify rural populations as a key priority for targeted educational and screening interventions. Across both settings, educational attainment remains a consistent determinant of cancer awareness.

Family income was not significantly associated with knowledge of warning signs, consistent with Batra et al. [29], but in contrast to Hvidberg et al. [39], who reported better knowledge of cancer warning signs among individuals with higher incomes. This difference may reflect greater contact with healthcare providers and higher educational attainment among higher-income groups. Similarly, family income was not significantly associated with knowledge of cancer risk factors, consistent with Shi et al. [40]. However, their findings suggested that knowledge of some risk factors, such as alcohol consumption and processed meat intake, remained limited even among higher-income families. In our study, family income was significantly associated with general cancer knowledge, consistent with Hvidberg et al. [39]. It was also significantly associated with overall cancer knowledge, consistent with Azimi et al. [41], who reported that higher family income was associated with better knowledge of oral cancer.

Regarding specific risk factors, only 13.5% of respondents correctly identified eating genetically modified (GM) foods as not a cancer risk factor. While the validated assessment instrument adapted for this study includes GM foods to test for widespread public misconceptions [19, 20], extensive scientific consensus currently classifies this belief as a common public myth rather than an established modifiable risk factor (such as tobacco use or obesity) [42]. Consequently, the low proportion of correct responses on this item indicates a widespread adherence to this myth among the participants, rather than a true deficit in recognizing established health risks. Future cancer awareness instruments should continue to carefully differentiate between established, evidence-based modifiable risk factors and common public myths to accurately gauge public health literacy and target educational campaigns toward dispelling these misconceptions.

Having a chronic illness was not associated with knowledge of cancer warning signs, in contrast to Salika et al. [43], who suggested that individuals aged over 50 years with chronic conditions such as hypercholesterolemia or hypertension may be more likely to seek medical advice when they notice potential alarm symptoms, such as an unexplained cough. However, this association is not straightforward, because several chronic diseases may present with symptoms that overlap with cancer warning signs, making it difficult for patients to distinguish between them and potentially delaying help-seeking. Similarly, we found no significant association between having a chronic illness and knowledge of cancer risk factors. To our knowledge, the literature on how chronic illness influences cancer knowledge remains limited, as most studies have examined help-seeking behavior rather than knowledge itself. Nevertheless, individuals with chronic illness and insufficient cancer knowledge may hold less favorable attitudes toward screening [44]. Although multimorbidity has been linked to an increased risk of cancer [45], this reflects disease risk rather than knowledge of cancer-related warning signs or risk factors.

In our study, more than half of the participants reported the internet as their main source of information. This finding is consistent with previous studies highlighting the growing reliance on online platforms for cancer-related information [25, 46, 47]. This pattern may reflect the increasing accessibility of digital technologies, the broad age distribution of our sample, and the predominance of participants aged 18–44 years, who are among the most frequent internet users. It may also be influenced by the limited time available for physician–patient interactions, prompting individuals to seek health information online [48]. Our findings further suggest that the internet plays an important role in cancer awareness, as reflected by the relatively high proportion of correct responses across general knowledge, warning signs, and risk factors. This is in line with Prager et al. [49], who emphasized the importance of accessible information in strengthening cancer awareness. Nevertheless, given the need to improve health literacy and the variable reliability of online information, there is a clear need to strengthen communication between physicians and patients and to educate the public on identifying trustworthy medical sources online.

Educational level was significantly associated with differences across all cancer knowledge subdomains. Participants with higher education, particularly those with university and postgraduate qualifications, achieved higher median scores in all domains. In contrast, those with lower educational attainment, such as high school graduates and illiterate participants, generally scored lower. These findings indicate that higher educational attainment is strongly associated with better cancer-related knowledge, as supported by multiple studies. Abdelaziz et al. [50] showed that individuals with higher educational attainment had better knowledge of breast cancer warning signs and risk factors. Yehia and Alboraie [18] reported similar findings for colorectal cancer awareness. Amin et al. [51] found that postgraduate students had greater screening knowledge than undergraduates, and Ibraheim et al. [46] demonstrated that educational interventions improved nursing students’ awareness. However, Abdelmonsef Ahmed et al. [52] revealed that universities still fail to provide sufficient cancer prevention education. Collectively, these studies underscore the need to strengthen cancer education across all academic levels to improve awareness and promote early detection behaviors.

Participants with medical affiliations in our study attained higher median scores across all domains than those without medical associations. Egyptian studies consistently indicate that higher educational attainment and exposure to the medical field are associated with greater cancer awareness. Among university students, colorectal cancer research has shown that education improves knowledge and reduces barriers to screening [52]. Similarly, bladder cancer surveys have demonstrated that medical students and healthcare staff recognize symptoms and risk factors more accurately than non-medical groups [53]. Similarly, caregivers have been found to possess greater knowledge than non-caregivers, even at comparable educational levels [50]. At the same time, cervical cancer studies have shown that education and contact with healthcare services predict higher awareness and screening uptake [23]. Collectively, these findings suggest that exposure to medical education or clinical practice is consistently associated with better understanding of cancer risk factors, symptoms, and preventive behaviors.

### Strengths and limitations

This study possesses several notable strengths and limitations. A primary strength is the relatively large and diverse sample of 399 participants, encompassing a range of sexes, age groups, educational levels, and residency statuses. However, the findings are not fully generalizable to the broader population, as the study has a health facility-based, rather than community-based, design and used convenience sampling; a multicentric approach would have improved representativeness. As participants were recruited from individuals attending primary healthcare facilities, they may be more health-conscious or more frequently exposed to health information than the general adult population of Mansoura. Consequently, the awareness levels reported here should be interpreted as a likely upper-bound estimate, and true population-level awareness may be lower. The use of a self-administered questionnaire addressing general cancer knowledge, warning signs, and risk factors enabled a comprehensive assessment of community knowledge. Nonetheless, reliance on self-reported data may not accurately capture actual understanding and may introduce recall and social desirability bias. The cross-sectional design further restricts causal inference, as observed associations cannot establish temporality or directionality.

Furthermore, although a previously published questionnaire was used, it includes items designed to test for common public misconceptions—such as the belief that genetically modified (GM) foods cause cancer—alongside established modifiable risk factors. While evaluating susceptibility to these myths is valuable, aggregating recognition of scientifically proven risks (like tobacco use or obesity) with myth identification into a single overall knowledge score may complicate interpretation of participants' true health literacy. While most participants identified the internet and social media as their primary sources of cancer information, the study did not assess whether this knowledge led to preventive behaviors, such as screening uptake or lifestyle changes. Future research should, therefore, investigate not only sources of knowledge but also attitudes, intentions, and health-related practices.

## Conclusion

This study demonstrated that adults attending primary healthcare facilities in Mansoura possess relatively good general knowledge about cancer, particularly regarding the importance of regular medical check-ups and certain preventive behaviors. However, awareness of specific cancer warning signs and modifiable risk factors remains insufficient, as reflected by the moderate overall knowledge score and the limited recognition of several key symptoms and risk factors. Educational attainment and affiliation with the medical field were the strongest predictors of cancer knowledge, whereas sex and residency had no significant influence.

Targeted awareness programs focusing on warning signs, lifestyle-related risk factors, and prevention strategies are particularly needed for individuals with lower educational levels and those without medical backgrounds. Strengthening community-based cancer education may contribute to earlier diagnosis, improved preventive behaviors, and ultimately a reduced cancer burden in Egypt.

## Data Availability

All data are available on reasonable request from the corresponding author.

## Abbreviations

CA: Cancer Awareness
CT: Computed Tomography
HUE: Horus University Egypt
IRB: Institutional Review Board
Max: Maximum
Min: Minimum
N: Number (Sample Size)
NCI: National Cancer Institute
ORL: Otorhinolaryngology
Q1-Q3: Interquartile Range (25th to 75th percentiles)
SEER-CAHPS: Surveillance, Epidemiology, and End Results Consumer Assessment of Healthcare Providers and Systems
UAE: United Arab Emirates
WHO: World Health Organization
